# Advanced Human Immunodeficiency Virus Disease at Diagnosis in Mozambique and Swaziland

**DOI:** 10.1093/ofid/ofx156

**Published:** 2017-07-23

**Authors:** Stephanie A Kujawski, Matthew R Lamb, Maria Lahuerta, Margaret L McNairy, Laurence Ahoua, Fatima Abacassamo, Harriet Nuwagaba-Biribonwoha, Averie Gachuhi, Wafaa M El-Sadr, Batya Elul

**Affiliations:** 1 Department of Epidemiology, Columbia University Mailman School of Public Health, New York, New York; 2 ICAP at Columbia University Mailman School of Public Health, New York, New York; 3 Weill Cornell Medical College, New York, New York; 4 Centro de Colaboração em Saude, Maputo, Mozambique

**Keywords:** advanced HIV disease, late diagnosis, point-of-care CD4 testing, sub-Saharan Africa

## Abstract

**Background:**

Early diagnosis of human immunodeficiency virus (HIV) is a prerequisite to maximizing individual and societal benefits of antiretroviral therapy.

**Methods:**

Adults ≥18 years of age testing HIV positive at 10 health facilities in Mozambique and Swaziland received point-of-care CD4^+^ cell count testing immediately after diagnosis. We examined median CD4^+^ cell count at diagnosis, the proportion diagnosed with advanced HIV disease (CD4^+^ cell count ≤350 cells/μL) and severe immunosuppression (CD4^+^ cell count ≤100 cells/μL), and determinants of the latter 2 measures.

**Results:**

Among 2333 participants, the median CD4^+^ cell count at diagnosis was 313 cells/μL (interquartile range, 164–484), more than half (56.5%) had CD4^+^ ≤350 cells/μL, and 13.9% had CD4^+^ ≤100 cells/μL. The adjusted relative risk (aRR) of both advanced HIV disease and severe immunosuppression at diagnosis was higher in men versus women (advanced disease aRR = 1.31; 95% confidence interval [CI] = 1.16–1.48; severe immunosuppression aRR = 1.54, 95% CI = 1.17–2.02) and among those who sought HIV testing because they felt ill (advanced disease aRR = 1.30, 95% CI = 1.08–1.55; severe immunosuppression aRR = 2.10, 95% CI = 1.35–2.26). Age 18–24 versus 25–39 was associated with a lower risk of both outcomes (advanced disease aRR = 0.70, 95% CI = 0.59–0.84; severe immunosuppression aRR = 0.62, 95% CI = 0.41–0.95).

**Conclusions:**

More than 10 years into the global scale up of comprehensive HIV services, the majority of adults diagnosed with HIV at health facilities in 2 high-prevalence countries presented with advanced disease and 1 in 7 had severe immunosuppression. Innovative strategies for early identification of HIV-positive individuals are urgently needed.

Diagnosis of people living with human immunodeficiency virus (PLWH) when they have advanced disease (late diagnosis) increases their risk of morbidity and mortality [1–3], escalates the cost of their clinical management [4], and limits the potential of antiretroviral therapy (ART) to reduce onward transmission of human immunodeficiency virus (HIV) [5]. In sub-Saharan Africa, where the vast majority of PLWH reside, several studies have examined the prevalence and correlates of advanced disease at enrollment in HIV care and at ART initiation [6–9], but data on late diagnosis, a critical precursor to both late enrollment and late ART initiation, are particularly scant [3]. Because global HIV programs are increasingly focused on enhancing HIV testing coverage as a first step towards epidemic control [10, 11], examining immunological status at diagnosis can indicate the extent to which testing efforts are successfully identifying PLWH before disease progression so that individual and population benefits of ART can be maximized.

To date, only 5 studies have reported on the immunological status of adult PLWH at diagnosis in sub-Saharan Africa [12–16]. In those studies, median CD4^+^ cell count at diagnosis ranged from a 186–242 cells/μL among individuals tested at health facilities [12, 13] to 414 cells/μL among those tested in the community [14]. The proportion diagnosed with CD4^+^ cell counts ≤350 cells/μL ranged from 43% to 72% [14–16], and, of concern, in 1 study, 34% had CD4^+^ cell counts <100 cells/μL at the time of HIV testing [12]. Male sex, age, distance to a health facility, and poor emotional health emerged as determinants of late diagnosis in these studies [12, 15]. Because these studies were limited by small sample sizes [12], were conducted in urban areas in a single country [12, 15], and were conducted largely in the early stages of the global scale up of HIV programs [13, 15, 16], we used data from 2 recently completed implementation science studies that were conducted in rural and urban health facilities in Mozambique and Swaziland to provide a more robust assessment of the magnitude and predictors of late diagnosis.

## METHODS

### Study Setting and Population

We used data from the Engage4Health study in Mozambique and the Link4Health study in Swaziland [17, 18]. Both studies aimed to improve linkage to care after HIV diagnosis and sustained retention in HIV care among adults newly diagnosed with HIV in health facilities through combination intervention strategies targeting known barriers across the HIV care continuum. Ten primary health clinics in Mozambique and 10 primary- and secondary-level facilities in Swaziland were matched as study units, then randomized to implement either the standard of care or the combination intervention strategy. In both countries, point-of-care CD4^+^ cell count testing immediately after the HIV positive test was conducted at the sites assigned to the combination intervention strategy. This analysis is restricted to the 10 facilities/study units (5 facilities in Mozambique and 5 study units in Swaziland) that implemented the intervention strategy. Of the facilities/study units included in this analysis, half were located in rural areas. Detailed information on the study design and combination intervention strategies used in these studies is available elsewhere [17, 18].

### Data Collection

In Mozambique, individuals were recruited from voluntary counseling and testing (VCT) clinics within the study facilities, whereas in Swaziland, participants were recruited from VCT clinics and provider-initiated testing and counseling venues within the study facilities. After posttest counseling, HIV testing counselors referred newly diagnosed adults to study personnel for information on the study. Eligibility criteria included being ≥18 years of age, speaking the main local language (Portuguese or Xitsua in Mozambique; English or SiSwati in Swaziland), not currently pregnant, not planning to leave the community in the next 12 months, agreeing to be referred to HIV care services at the diagnosing health facility, and not having been enrolled in HIV care or initiated ART in the prior 6 months. As noted above, all study participants received point-of-care CD4^+^ cell count testing in the HIV testing clinic immediately after diagnosis using PIMA Analyser (Inverness). Participants completed a structured interview that gathered information on demographics, psychosocial factors, and HIV testing history, knowledge, and attitudes. Enrollment ran from April 2013 to June 2015 in Mozambique and from August 2013 to November 2014 in Swaziland.

### Ethical Approvals

Both studies were approved by the Columbia University Medical Center Institutional Review Board. In addition, the Engage4Health study was approved by the Mozambican National Committee for Bioethics in Health, and the Link4Health study was approved by the US Centers for Disease Control and Prevention and the Swaziland Scientific and Ethics Committee. All study participants provided written informed consent before study enrollment. The trials are registered at ClinicalTrials.gov: Engage4Health study number NCT01930084; Link4Health study number NCT01904994.

### Measures

Late diagnosis of HIV diseases included the following 2 categories. (1) Advanced HIV infection at diagnosis was defined as having CD4^+^ cell count ≤350 cells/μL, in alignment with national thresholds for treatment initiation in both countries at the time of study implementation [19, 20]. (2) Severe immunosuppression at diagnosis was defined as having a CD4^+^ cell count of ≤100 cells/μL.

Correlate selection was guided by the Aday and Anderson [21] model for healthcare access. Predisposing factors, defined as demographic and psychosocial factors and that may influence service use, included the following: sex; age (18–24 years, 25–39 years, 40+ years); education (≥secondary school, <secondary school); marital status (married and living together or not married but living together with 1 or more partners, married/have partner but not living together, single); HIV-related knowledge; and anticipated HIV-related stigma. Enabling factors, which reflect the means individuals have to access health services, included the following: residence location, which was determined by the location of the diagnosing health facility (urban, rural); household wealth (split into tertiles); distance from the participant’s home to the health facility (≤30 minutes, 31–60 minutes, >60 minutes); whether other members of the participant’s household were living with HIV (no one, family member, don’t know); whether the participant was away from home for an extended period in last 12 months; and whether the participant had confidants with whom they shared private matters (some, none). The need component, representing the degree of illness, was categorized based on the following: whether the participant was hospitalized in the prior 12 months (yes, no); the type of HIV testing received (voluntary, provider suggested); whether the participant had previously been tested for HIV; whether the participant was tested because s/he perceived him/herself to be at risk of HIV infection (eg, partner was sick, routinely gets tested), because s/he was influenced by others (eg, partner/friend suggested testing), and because s/he felt sick (eg, weight loss, sores). Reasons for testing variables were coded from a multiresponse question, with participants able to answer more than 1 reason why they received an HIV test. These reasons were then grouped into categories as a dichotomous variable (yes, no).

For HIV knowledge, correct responses to 8 questions from the 2 studies were summed to create a score, which was then dichotomized at the median into high and low HIV knowledge. Anticipated stigma was assessed using 5 questions from the Link4Health and Engage4Health studies following the concept described by Earnshaw and Chaudoir [22]. In the Link4Health study, each of the questions had 4 response options ranging from “strongly agree” to “strongly disagree”, which were dichotomized into “agree” and “disagree” and then summed to create a score. In the Engage4Health study, 2 response categories, (agree and disagree) were included, and responses were similarly summed to create a score. The scores were combined across studies and split at the median into high stigma and low stigma. To measure household wealth, we used principal component analysis drawing on 4 asset variables in the 2 studies, and then we divided this metric into tertiles.

### Statistical Analysis

We examined CD4^+^ cell count distribution at diagnosis for the total sample, by country, and by sex as continuous and categorical variables using Wilcoxon-Mann-Whitney tests and χ^2^ tests, respectively. To assess factors associated with advanced disease and severe immunosuppression at diagnosis, we used log-Poisson relative risk (RR) regression with empirical standard error estimation and random-intercepts to account for clustering by health facility. Variables that were significant at *P* < .20 in bivariate analyses were included in multivariable models. Sex-specific and country-specific models were then run per outcome. Regression models used complete case analysis, which included 97.2% of all study participants. Statistical analyses were conducted using SAS, version 9.4 (SAS Institute, Cary, NC).

## RESULTS

A total of 2333 participants were enrolled at the 10 health facilities included in this analysis: 1237 (53.0%) in Mozambique and 1096 (47.0%) in Swaziland (Table 1). Participants averaged 34.3 years (standard deviation = 10.5) of age, and the majority resided in urban areas (59.6%) and were female (63.0%). Most participants (83.2%) reported that they had voluntarily sought HIV testing, and many did so because they perceived themselves to be at risk of HIV infection (39.4%) or were ill (54.0%). More than one third (38.7%) of participants had been tested for HIV previously, and 32.5% reported that another member in their household was living with HIV. The majority (77.1%) of participants had low levels of HIV knowledge, and approximately half (48.9%) anticipated high levels of stigma after diagnosis, with this percentage reaching 57.9% in Mozambique.

**Table 1.** Baseline Characteristics of Participants Receiving a Point-of-Care CD4+ Cell Count Test at HIV Diagnosis in 10 Health Facilities in Mozambique and Swaziland (N = 2333)

Participant Characteristics	Total Sample						Mozambique		Swaziland	
	Total Sample (N = 2333)		Males (N = 871)		Females (N = 1462)		Engage4Health (N = 1237)		Link4Health (N = 1096)	
	N	%	N	%	N	%	N	%	N	%
CD4, median (IQR)	313	164–484	253	129–406	353	197–518	315	176–497	311	158.5–472.5
Facility Location										
Urban	1391	59.6	525	60.3	866	59.2	671	54.3	720	65.7
Rural	942	40.4	346	39.7	596	40.8	566	45.7	376	34.3
Sex										
Male	871	37.3	-	-	-	-	428	34.6	443	40.4
Female	1462	62.7	-	-	-	-	809	65.4	653	59.6
Age, mean (SD)	34.3	10.5	36.6	10.5	33.0	10.2	34.4	9.8	34.2	11.2
18–24	369	15.8	60	6.9	309	21.2	159	12.9	210	19.2
25–39	1354	58.1	551	63.3	803	55.0	742	60.0	612	55.9
40+	609	26.1	260	29.9	349	23.9	336	27.2	273	24.9
Education										
≥Secondary school	1063	45.6	412	47.3	651	44.6	446	36.1	617	56.4
<Secondary school	1268	54.4	459	52.7	809	55.4	790	63.9	478	43.7
Marital Status										
Married and living together, or not married but living together with 1 or more partners	1033	44.4	490	56.3	543	37.2	631	51.1	402	36.8
Married/have partner but not living together	692	29.7	227	26.1	465	31.9	187	15.1	505	46.2
Currently single	604	25.9	153	17.6	451	30.9	418	33.8	186	17.0
Knowledge score (0–8), mean (SD)	6.4	1.4	6.4	1.5	6.4	1.4	6.2	1.5	6.6	1.3
High knowledge (8)	534	22.9	223	25.6	311	21.3	218	17.6	316	28.8
Low knowledge (0–7)	1799	77.1	648	74.4	1151	78.7	1019	82.4	780	71.2
Stigma score (0–5), mean (SD)	2.6	1.4	2.6	1.4	2.6	1.4	2.7	1.4	2.4	1.4
High stigma (3–5)	1141	48.9	423	48.6	718	49.1	704	57.9	437	39.9
Low stigma (0–2)	1191	51.1	448	51.4	743	50.9	533	43.1	658	60.1
Wealth Index										
Tertile 1	817	35.1	304	35.0	513	35.1	330	26.8	487	44.4
Tertile 2	743	31.9	270	31.1	473	32.4	332	26.9	411	37.5
Tertile 3	769	33.0	295	34.0	474	32.5	571	46.3	198	18.1
Distance From Home to Health Facility										
≤30 minutes	1247	54.5	486	56.3	761	53.4	557	46.2	690	63.8
31–60 minutes	764	33.4	289	33.5	475	33.4	434	36.0	330	30.5
61+ minutes	276	12.1	88	10.2	188	13.2	214	17.8	62	5.7
Others in Household Living With HIV										
No one	1193	51.1	434	49.8	759	51.9	580	46.9	613	55.9
Other family member	758	32.5	301	34.6	457	31.3	331	26.8	427	39.0
Don’t know	382	16.4	136	15.6	246	16.8	326	26.4	56	5.1
No. Times Away From Home in last 12 Months										
0	1783	76.6	631	72.7	1152	79.0	867	70.4	916	83.7
1+	544	23.4	237	27.3	307	21.0	365	29.6	179	16.4
Have Confidants With Whom Share Private Matters										
None	496	21.3	176	20.2	320	21.9	116	9.4	380	34.7
Some	1836	78.7	695	79.8	1141	78.1	1121	90.6	175	65.3
No. of Times Hospitalized in Last 12 Months										
0	2209	94.7	827	95.0	1382	94.5	1178	95.2	1031	94.1
1+	124	5.3	44	5.1	80	5.5	59	4.8	65	5.9
Type of HIV Test										
Voluntary	1942	83.2	715	82.1	1227	83.9	1007	81.4	935	85.3
Provider suggested	391	16.8	156	17.9	235	16.1	230	18.6	161	14.7
Previously Tested for HIV										
First time testing	1430	61.3	584	67.1	846	57.9	788	63.7	642	58.6
Previously tested for HIV	902	38.7	286	32.9	616	42.1	449	36.3	453	41.4
If Previously Tested, First Time Positive Result (N = 902)										
Yes	693	76.8	213	74.5	480	77.9	368	82.0	325	71.7
No	209	23.2	73	25.5	136	22.1	81	18.0	128	28.3
Years since first positive test result (N = 182), mean (SD)	1.9	2.5	1.7	2.4	2.0	2.5	0.6	1.0	2.4	2.7
<1 year	98	46.9	34	46.6	64	47.1	45	55.7	53	41.4
≥1–2 years	31	14.8	12	16.4	19	14.0	8	9.9	23	18.0
≥2–3 years	12	5.7	5	6.9	7	5.2	1	1.2	11	8.6
3+ years	41	19.6	10	13.7	31	22.8	2	2.5	39	30.5
Missing	27	12.9	12	16.4	15	11.0	25	30.9	2	1.6
Reason for testing: risk perception	918	39.4	330	37.9	588	40.3	399	32.3	519	47.5
Reason for testing: influenced by others	234	10.0	90	10.3	144	9.9	184	14.9	50	4.6
Reason for testing: felt sick/illness	1259	54.0	499	57.3	760	52.1	626	50.6	633	57.9

Abbreviations: HIV, human immunodeficiency virus; IQR, interquartile range; SD, standard deviation.

**Figure 1.**
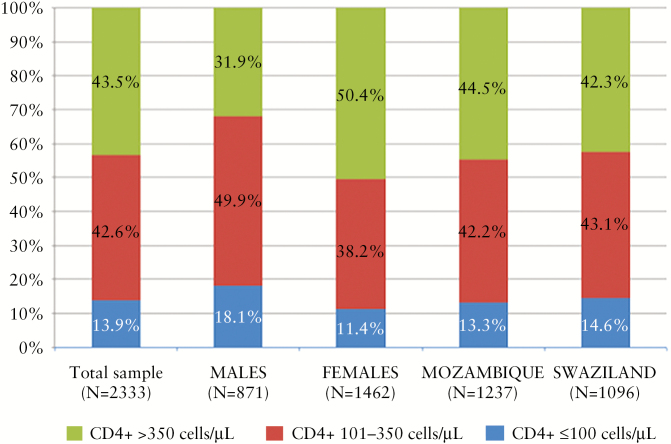
CD4^+^ cell count distribution of participants at human immunodeficiency virus (HIV) diagnosis in 10 facilities in Mozambique and Swaziland (N = 2333).

The median CD4^+^ cell count at diagnosis was 313 cells/μL (interquartile range [IQR], 164–484) and was lower among men compared with women (men: 253 cells/μL [IQR, 129–406] and women: 353 cells/μL [IQR, 197–518], *P* ≤ .0001). The median CD4^+^ cell count among participants in Mozambique was 315 cells/μL (IQR, 176–497) and 311 cells/μL in Swaziland (IQR, 158.5–472.5), *P* = .10 (Table 1). As shown in Figure 1, at the time of diagnosis, more than half (56.5%) of the participants had advanced disease (CD4^+^ ≤350 cells/μL), and 13.9% were severely immunosuppressed (CD4^+^ ≤100 cells/μL). A higher percentage of males (68.1%) compared with females (49.6%) had CD4^+^ ≤350 cells/μL (*P* < .0001) and CD4^+^ ≤100 cells/μL (males 18.1% vs females 11.4%, *P* < .0001) at the time of diagnosis, but no significant differences were observed by country (advanced disease: Mozambique 55.5% vs Swaziland 57.7%, *P* = .30; severe immunosuppression: Mozambique 13.3% vs Swaziland 14.6%, *P* = .37).

Table 2 presents results from bivariate and multivariable regression models examining factors associated with advanced HIV disease and severe immunosuppression at diagnosis. In adjusted models, men were significantly more likely to be diagnosed late than women (advanced disease: RR = 1.31, 95% CI = 1.16–1.48; severe immunosuppression: RR = 1.54, 95% CI = 1.17–2.02). Participants who reported seeking HIV testing because they felt sick were significantly more likely to have advanced disease (RR = 1.30; 95% CI, 1.08–1.55) and have severe immunosuppression (RR = 2.10; 95% CI, 1.35–2.26). Participants with higher HIV knowledge were also at significantly higher risk of being diagnosed when they were severely immunosuppressed (RR = 1.35; 95% CI, 1.03–1.78). Young adults age 18–24 years were significantly less likely to have advanced disease at diagnosis (RR = 0.70; 95% CI, 0.59–0.84) and less likely to have severe immunosuppression (RR = 0.62; 95% CI, 0.41–0.95) than those 25–39 years of age. In addition, results were similar when the analysis was stratified by sex and country, with 1 additional predictor of note emerging (Supplemental Tables 1 and 2): in Mozambique, participants reporting that another household member(s) was living with HIV were less likely to be diagnosed with severe immunosuppression (RR = 0.58; 95% CI, 0.36–0.94).

**Table 2.** Relative Risk Regression Models of Advanced HIV Disease at Diagnosis (CD4^+^ ≤350) and Severe Immunosuppression at Diagnosis (CD4^+^ ≤100) in 10 Health Facilities in Mozambique and Swaziland (N = 2267)

Participant Characteristics	N	%Advanced HIV Disease	Advanced HIV Disease (CD4 ≤350)				%Severe Immunosuppressed	Severe Immunosuppression (CD4 ≤100)			
			Bivariate		Multivariable			Bivariate		Multivariable	
			RR	95% CI	RR	95% CI		RR	95% CI	RR	95% CI
Male (vs female)	857	67.9	1.39	1.24–1.57	1.31	1.16–1.48	18.1	1.59	1.24–2.03	1.54	1.17–2.02
Ages 25–39 (ref)	1309	58.5					15.0				
Ages 18–24	363	36.6	0.63	0.53–0.74	0.70	0.59–0.84	7.7	0.52	0.35–0.79	0.62	0.41–0.95
Ages 40+	595	62.2	1.07	0.96–1.18	1.01	0.90–1.13	15.1	0.99	0.76–1.30	0.90	0.67–1.22
Secondary education (vs none/ primary)	1040	54.0	0.93	0.84–1.04	1.00	0.88–1.13	13.8	0.98	0.79–1.22		
Married/partner and living together (ref)	1011	57.2					12.7				
Married/partner not living together	676	51.3	0.88	0.76–1.01	0.95	0.81–1.12	12.1	0.92	0.64–1.30	0.99	0.71–1.37
Single	580	59.3	1.04	0.90–1.20	1.07	0.93–1.23	17.9	1.43	1.00–2.05	1.42	0.98–2.07
High HIV knowledge (vs low)	524	57.6	1.04	0.89–1.22			16.6	1.29	1.03–1.62	1.35	1.03–1.78
High HIV stigma (vs low)	1110	56.8	1.03	0.89–1.19			14.6	1.09	0.83–1.43		
Wealth index tertile 1 (ref)	798	55.4					13.7				
Tertile 2	720	58.2	1.05	0.93–1.18			14.6	1.08	0.74–1.57		
Tertile 3	749	54.5	0.99	0.86–1.14			13.4	1.04	0.77–1.39		
Distance to health facility ≤30 minutes (ref)	1235	54.5					13.8				
Distance 31–60 minutes	757	57.2	1.06	0.94–1.19			13.6	1.01	0.76–1.34		
Distance 61+ minutes	275	59.3	1.10	0.91–1.32			14.9	1.09	0.71–1.67		
No household member with HIV (ref)	1149	58.1					14.8				
Household member with HIV	743	51.7	0.89	0.79–1.01	0.93	0.82–1.06	10.5	0.71	0.49–1.04	0.86	0.60–1.25
Don’t know	375	57.9	1.00	0.86–1.15	0.97	0.84–1.11	17.6	1.14	0.78–1.67	1.07	0.73–1.56
Away from home in last 12 months (vs none)	534	58.2	1.05	0.94–1.17			14.8	1.05	0.82–1.34		
Has confidants (vs none)	1782	54.8	0.91	0.79–1.05	0.91	0.79–1.05	13.9	1.05	0.64–1.71		
Hospitalized in last 12 months (vs none)	119	57.1	1.02	0.78–1.34			15.1	1.07	0.66–1.74		
Voluntary testing (vs provider suggested)	1881	54.8	0.89	0.76–1.04	0.93	0.78–1.12	12.8	0.71	0.46–1.12	0.77	0.49–1.19
Participant previously tested for HIV (vs no)	880	49.3	0.82	0.72–0.92	0.90	0.80–1.03	13.1	0.90	0.65–1.25		
Reason for testing: risk perception	893	49.4	0.82	0.71–0.96	0.97	0.81–1.17	9.0	0.53	0.40–0.71	0.80	0.56–1.13
Reason for testing: influenced by others	230	52.6	0.94	0.75–1.17			6.1	0.43	0.22–0.84	0.59	0.32–1.08
Reason for testing: felt sick/ illness	1221	64.6	1.41	1.22–1.63	1.30	1.08–1.55	19.5	2.65	1.67–4.22	2.10	1.35–3.26

Abbreviations: CI, confidence interval; HIV, human immunodeficiency virus; ref, reference; RR, relative risk.

## DISCUSSION

In this large study of adults newly diagnosed with HIV in diverse health facilities in Mozambique and Swaziland—2 countries with significant HIV epidemics (11% prevalence in Mozambique [23], 32% prevalence in Swaziland [24]) and very low HIV testing coverage (9%–14% of males and 22%–26% of females [25] in the prior 12 months)—more than half the participants (56.5%) had CD4^+^ cell counts ≤350 cells/μL, making them eligible for ART at the time of diagnosis, according to prevailing national treatment guidelines. Furthermore, 13.9% had severe immune suppression (CD4^+^ cell counts ≤100 cells/μL) when diagnosed. These findings underscore the critical need to promote HIV testing and expand testing coverage using novel testing approaches in diverse settings to ensure opportunities for earlier diagnosis and treatment initiation. Importantly, late diagnosis and ART initiation limit the potential individual and population benefits of reducing morbidity, mortality, and onward transmission, which are key to the control of the HIV epidemic and the sustainability of HIV prevention and treatment efforts to date [1–3, 5].

Three studies conducted in sub-Saharan Africa measured median CD4^+^ cell count at the time of HIV diagnosis and reported results ranging from 186 cells/μL to 414 cells/μL [12–14], potentially reflecting differences in countries, populations (urban vs rural), testing venues (community-based testing vs testing in health facilities), and testing approaches (eg, VCT vs provider-initiated testing and counseling). For example, the highest median CD4^+^ cell count at diagnosis (414 cells/μL) was observed among individuals diagnosed via community-based testing in South Africa [14], arguably a healthier population of PLWH than those seeking out testing at health facilities. Furthermore, when compared with prior studies, our results suggest modest potential progress in the proportion of PLWH diagnosed with advanced disease over time. Indeed, in a study conducted from 2005 to 2008, 72% of individuals newly diagnosed with HIV at a health facility in Nigeria had CD4^+^ cell counts ≤350 cells/μL [16] compared with 56% in our study. This trend is consistent with temporal improvements in CD4^+^ cell counts at enrollment in HIV care and at ART initiation reported in 2 studies using programmatic data from 4 sub-Saharan African countries [6, 7]. At the same time, a recent meta-analysis of studies from sub-Saharan Africa found no significant change in CD4^+^ at enrollment in care (N = 56 studies) or at ART initiation (N = 71 studies) from 2002 to 2013 [26], suggesting that further improvements in CD4^+^ cell count at diagnosis will be needed before any meaningful downstream effects are observed.

We also identified several factors that represent potentially important areas of intervention to promote timely diagnosis. Consistent with prior research [12, 15], male sex emerged as an important determinant of advanced disease and particularly of severe immunosuppression at diagnosis, likely reflecting sex differentials in health-seeking behaviors [27, 28]. Testing campaigns explicitly targeting men may be effective in early diagnosis of HIV among men. Similar to previous studies, we also found an increased likelihood of advanced disease and severe immunosuppression at diagnosis among participants who reported seeking HIV testing because they felt sick or were ill [15, 29]. Increasing knowledge of HIV risk and of the need for routine HIV testing in high prevalence settings as well as expanding access to testing will be critical to identifying PLWH before evidence of immunosuppression emerges. We were surprised to find that in our study, participants with higher knowledge of HIV were more likely to be diagnosed with severe immunosuppression. Given the cross-sectional nature of this analysis, we cannot rule out that individuals with symptoms received information regarding the disease before seeking HIV testing—either on their own initiative, as a result of prior HIV testing, or from family members living with HIV.

Several findings also point to potential successes with regards to HIV testing programs. In Mozambique, participants living in households with other PLWH were less likely to have severe immunosuppression at the time of diagnosis, possibly as a result of family-focused approaches to HIV testing [30]. In addition, higher anticipated stigma was not significantly associated with advanced disease or severe immunosuppression at diagnosis, possibly reflecting the reduced influence of stigma in HIV testing [31, 32], although this finding should be further investigated in studies with more refined measures of stigma.

This study has a number of strengths. First, it is one of the few, and the largest to date, to explore the magnitude and predictors of advanced stage HIV disease and severe immunosuppression at the time of diagnosis. In addition, the study included participants from diverse types of health facilities situated in both rural and urban settings and from 2 countries with disparate health systems, providing a diverse sample. We also included a variety of demographic, psychosocial, and clinical measures when examining predictors of late diagnosis, capturing key aspects of the Aday and Anderson [21] healthcare access model. A few limitations should also be noted. The cross-sectional nature of the participant interview relative to outcome measurement makes it difficult to ensure temporality of some exposures in relation to the outcome. Furthermore, study inclusion and exclusion criteria temper our ability to generalize our findings to all adults diagnosed in health facilities.

## CONCLUSIONS

More than 10 years into the scale up of comprehensive HIV services in sub-Saharan Africa, this analysis illustrates (1) the persistent challenge of late diagnosis and (2) the critical need to capture PLWH earlier in their disease progression to ensure individual- and population-level benefits of ART. With current recommendations supporting treatment of all PLWH, this study highlights the importance of expanding testing efforts to reach all individuals with undiagnosed HIV [11]. At the same time, targeted efforts are needed to reach those individuals with advanced HIV disease to enable prompt initiation of ART for their own and for societal benefits of treatment.

## Supplementary Data

Supplementary materials are available at *Open Forum Infectious Diseases* online. Consisting of data provided by the authors to benefit the reader, the posted materials are not copyedited and are the sole responsibility of the authors, so questions or comments should be addressed to the corresponding author.

## Supplementary Material

ofx156_suppl_Supplementary_Tables1_2
