# Erratum

**DOI:** 10.1093/ofid/ofx191

**Published:** 2018-01-30

“Advanced HIV disease at diagnosis in Mozambique and Swaziland” by Kujawski et al. [Open Forum Infect Dis **2017**; doi: 10.1093/ofid/ofx156] is an Open Access article and should have contained the following copyright line:

© The Author [2018]. Published by Oxford University Press [on behalf of Infectious Diseases Society of America]. This is an Open Access article distributed under the terms of the Creative Commons Attribution License (http://creativecommons.org/licenses/by/4.0/), which permits unrestricted reuse, distribution, and reproduction in any medium, provided the original work is properly cited.

The authors regret these error.

