# Supplementary material for: Advanced Human Immunodeficiency Virus Disease at Diagnosis in Mozambique and Swaziland
Source: Open Forum Infect Dis. 2017 Jul 23;4(3):ofx156. doi: 10.1093/ofid/ofx156 (PMC5610724; doi:10.1093/ofid/ofx156)
Supplement: ofx156_suppl_Supplementary_Tables1_2 [file ofx156_suppl_supplementary_tables1_2.docx]

| **Supplemental Table 1: Relative risk regression models of advanced HIV disease at diagnosis (CD4+ ≤350) in 10 health facilities in Mozambique and Swaziland by sex and by country (N=2267)** | | | | | | | | |
| --- | --- | --- | --- | --- | --- | --- | --- | --- |
|  | **Males (N=857)** | | **Females (N=1410)** | | **Mozambique (N=1197)** | | **Swaziland (N=1070)** | |
|  | **Multivariable** | | **Multivariable** | | **Multivariable** | | **Multivariable** | |
|  | **RR** | **95% CI** | **RR** | **95% CI** | **RR** | **95% CI** | **RR** | **95% CI** |
| Male (vs. female) | - | - | - | - | 1.22 | 1.10-1.36 | 1.38 | 1.25-1.53 |
| Ages 25-39 (ref) |  |  |  |  |  |  |  |  |
| Ages 18-24 | 0.84 | 0.58-1.22 | 0.68 | 0.55-0.86 | 0.67 | 0.54-0.84 | 0.74 | 0.61-0.88 |
| Ages 40+ | 0.99 | 0.84-1.17 | 1.03 | 0.84-1.25 | 1.03 | 0.92-1.15 | 1.01 | 0.91-1.13 |
| Secondary education (vs. none/primary) | 0.98 | 0.83-1.15 | 1.02 | 0.86-1.21 | 0.98 | 0.88-1.10 | 1.00 | 0.91-1.11 |
| Married/partner and living together (ref) |  |  |  |  |  |  |  |  |
| Married/partner not living together | 1.03 | 0.86-1.24 | 0.87 | 0.69-1.10 | 0.80 | 0.66-0.96 | 0.99 | 0.89-1.12 |
| Single | 1.03 | 0.85-1.26 | 1.05 | 0.87-1.27 | 1.02 | 0.91-1.15 | 1.15 | 1.00-1.31 |
| No household member with HIV (ref) |  |  |  |  |  |  |  |  |
| Household member with HIV | 0.89 | 0.75-1.06 | 0.96 | 0.80-1.15 | 0.97 | 0.85-1.11 | 0.90 | 0.81-1.01 |
| Don't know | 0.89 | 0.72-1.10 | 1.01 | 0.81-1.27 | 1.04 | 0.90-1.19 | 0.90 | 0.71-1.14 |
| Has confidants (vs. none) | 0.94 | 0.75-1.17 | 0.88 | 0.74-1.05 | 0.94 | 0.79-1.12 | 0.90 | 0.81-1.00 |
| Voluntary testing (vs. provider suggested) | 0.94 | 0.72-1.22 | 0.93 | 0.72-1.20 | 0.96 | 0.83-1.07 | 0.85 | 0.75-0.94 |
| Participant previously tested for HIV (vs. no) | 0.91 | 0.76-1.09 | 0.91 | 0.75-1.10 | 0.94 | 0.84-1.05 | 0.84 | 0.75-0.94 |
| Reason for testing: risk perception | 1.06 | 0.86-1.32 | 0.91 | 0.71-1.16 | 0.93 | 0.81-1.07 | 1.02 | 0.91-1.15 |
| Reason for testing: felt sick/illness | 1.19 | 0.97-1.47 | 1.39 | 1.09-1.77 | 1.22 | 1.07-1.39 | 1.32 | 1.16-1.50 |
| Variables included in multivariable model have p<0.20 in bivariate regression | | | | | |  |  |  |

| **Supplemental Table 2: Relative risk regression models of severe immunosuppression at diagnosis (CD4+ ≤100) in 10 health facilities in Mozambique and Swaziland by sex and by country (N=2267)** | | | | | | | | |
| --- | --- | --- | --- | --- | --- | --- | --- | --- |
|  | **Males (N=857)** | | **Females (N=1410)** | | **Mozambique (N=1197)** | | **Swaziland (N=1070)** | |
|  | **Multivariable** | | **Multivariable** | | **Multivariable** | | **Multivariable** | |
|  | **RR** | **95% CI** | **RR** | **95% CI** | **RR** | **95% CI** | **RR** | **95% CI** |
| Male (vs. female) | - | - | - | - | 1.52 | 1.12-2.05 | 1.48 | 1.10-1.99 |
| Ages 25-39 (ref) |  |  |  |  |  |  |  |  |
| Ages 18-24 | 0.73 | 0.35-1.51 | 0.58 | 0.36-0.94 | 0.77 | 0.45-1.32 | 0.53 | 0.32-0.90 |
| Ages 40+ | 1.04 | 0.69-1.57 | 0.77 | 0.48-1.23 | 1.02 | 0.74-1.40 | 0.74 | 0.52-1.05 |
| Married/partner and living together (ref) |  |  |  |  |  |  |  |  |
| Married/partner not living together | 0.97 | 0.62-1.51 | 1.02 | 0.62-1.68 | 0.83 | 0.50-1.38 | 1.01 | 0.72-1.42 |
| Single | 1.35 | 0.88-2.08 | 1.50 | 0.79-2.86 | 1.22 | 0.89-1.67 | 1.82 | 1.25-2.66 |
| High HIV knowledge (vs. low) | 1.40 | 0.91-2.17 | 1.31 | 0.86-2.01 | 1.22 | 0.85-1.74 | 1.52 | 1.13-2.04 |
| No household member with HIV (ref) |  |  |  |  |  |  |  |  |
| Household member with HIV | 0.79 | 0.49-1.26 | 0.94 | 0.60-1.49 | 0.58 | 0.36-0.94 | 1.04 | 0.76-1.42 |
| Don't know | 0.98 | 0.65-1.50 | 1.13 | 0.65-1.98 | 1.00 | 0.70-1.43 | 0.84 | 0.37-1.88 |
| Voluntary testing (vs. provider suggested) | 0.78 | 0.45-1.33 | 0.76 | 0.42-1.39 | 0.74 | 0.52-1.06 | 0.97 | 0.64-1.48 |
| Reason for testing: risk perception | 0.98 | 0.62-1.56 | 0.63 | 0.34-1.14 | 0.69 | 0.44-1.09 | 0.77 | 0.52-1.15 |
| Reason for testing: influenced by others | 0.65 | 0.22-1.92 | 0.50 | 0.17-1.49 | 0.59 | 0.32-1.09 | 0.64 | 0.22-1.88 |
| Reason for testing: felt sick/illness | 1.97 | 1.06-3.67 | 2.15 | 0.94-4.90 | 1.57 | 1.06-2.33 | 2.56 | 1.58-4.16 |
| Variables included in multivariable model have p<0.20 in bivariate regression | | | | |  |  |  |  |
